# Metal ion levels and functional results after either resurfacing hip arthroplasty or conventional metal-on-metal hip arthroplasty: Short-term outcome of a randomized controlled trial

**DOI:** 10.3109/17453674.2011.625533

**Published:** 2011-11-24

**Authors:** José MH Smolders, Annemiek Hol, Willard J Rijnberg, Job LC van Susante

**Affiliations:** Department of Orthopaedics, Hospital Rijnstate, Arnhem, the Netherlands

## Abstract

**Background:**

Modern metal-on-metal hip resurfacing was introduced as a bone-preserving method of joint reconstruction for young and active patients; however, the large diameter of the bearing surfaces is of concern for potentially increased metal ion release.

**Patients and methods:**

71 patients (< 65 years old) were randomly assigned to receive either a resurfacing (R) hip arthroplasty (n = 38) or a conventional metal-on-metal (C) hip arthroplasty (n = 33). Functional outcomes were assessed preoperatively and at 6, 12, and 24 months. Cobalt and chromium blood levels were analyzed preoperatively and at 3, 6, 12, and 24 months.

**Results:**

All functional outcome scores improved for both groups. At 12 and 24 months, the median UCLA activity score was 8 in the R patients and 7 in the C patients (p < 0.05). At 24 months, OHS was median 16 in C patients and 13 in R patients (p < 0.05). However, in spite of randomization, UCLA scores also appeared to be higher in R patients at baseline. Satisfaction was similar in both groups at 24 months. Cobalt concentrations were statistically significantly higher for R patients only at 3 and 6 months. Chromium levels remained significantly higher for R patients until 24 months. No pseudotumors were encountered in either group. One R patient was revised for early aseptic loosening and in 2 C patients a cup insert was exchanged for recurrent dislocation.

**Interpretation:**

R patients scored higher on UCLA, OHS, and satisfaction at some time points; however, as for the UCLA, preoperative levels were already in favor of R. The differences, although statistically significant, were of minor clinical importance. Chromium blood levels were statistically significantly higher for R patients at all follow-up measurements, whereas for cobalt this was only observed up to 6 months. The true value of resurfacing hip arthroplasty over conventional metal-on-metal hip arthroplasty will be determined by longer follow-up and a possible shift of balance between their respective (dis)advantages.

Hip resurfacing arthroplasty has been proposed as the best treatment option for advanced osteoarthritis of the hip in young and active patients, with a component survivorship of 94% to 99.8% at 3 to 8 years of follow-up (Vail et al. 2006, Amstutz et al. 2007). In comparison to conventional total hip arthroplasty, it has been claimed that resurfacing has several advantages including preservation of femoral bone stock, better functional outcomes, and lower rates of dislocation (Vail et al. 2006, Lavigne et al. 2010, Smolders et al. 2010). There is, however, very little evidence available to support these claims. Only a limited number of studies have been published in which the clinical results of resurfacing and a conventional hip arthroplasty were compared. Most of these studies were matched cohort series, since true randomized controlled trials (RCT) are difficult to perform. There has been only one RCT where the clinical results of resurfacing arthroplasty were compared with the clinical results of a 28-mm metal-on-metal hip arthroplasty (Vendittoli et al. 2006). Both in that RCT and in some of the matched cohort series, a statistically significantly better functional score was found in selected outcome parameters for resurfacing at short-term follow-up; however, the clinical relevance is argued by the authors (Pollard et al. 2006, Vail et al. 2006, Fowble et al. 2009, Mont et al. 2009). In addition, the higher level of activity encountered for resurfacing hip prostheses in some series applies to both pre-and postoperative values (Pollard et al. 2006, Fowble et al. 2009, Mont et al. 2009). Another confounding factor, especially in the matched cohort series, may also have been the profound implant preference in the resurfacing group—which may have biased the eventual functional outcome relative to that of a conventional hip arthroplasty group (Mont et al. 2009, Bisseling et al. 2011).

In contrast to the potential advantage of a resurfacing, there is increasing concern about the possible toxic effects of focal and systemic metal ion exposure from these implants. It is well recognized that hip arthroplasties with a metal-on-metal bearing lead to an increase in blood levels of metal ions, especially cobalt and chromium (Back et al. 2005, Antoniou et al. 2008, Moroni et al. 2008, Garbuz et al. 2010). High wear from metal bearings may cause severe complications such as pseudotumor formation and soft tissue necrosis (Shimmin et al. 2005, Keegan et al. 2007, Pandit et al. 2008). In general, one would expect higher release of cobalt and chromium from resurfacing, with a relatively large articulating metal-on-metal bearing, as compared to a regular metal-on-metal hip arthroplasty, but there have been no conclusions in the literature about this issue (Smith et al. 2001).

In the only RCT that has been published (from Canada), clinical results of resurfacing were compared to those from a conventional 28-mm metal-on-metal hip arthroplasty (Vendittoli et al. 2006, 2010). Since the amount of metal ion release may play an important role in the eventual outcome and revision rate of an implant, we decided to perform a similar trial comparing resurfacing with a conventional 28-mm metal-on-metal hip arthroplasty. In contrast to the earlier trial (Vendittoli et al. 2010), besides functional outcome we also assessed the blood levels of cobalt and chromium for both implants with time. In this RCT, we questioned whether the functional results of resurfacing would indeed be superior to a conventional metal-bearing hip arthroplasty and whether a large-diameter resurfacing bearing would induce more release of metal ions than a similar but relatively small 28-mm bearing. As there is currently a lot of debate in the literature about the potential (dis)advantages of resurfacing, we felt that it would be appropriate to present the data of our exploratory RCT after short-term follow-up.

## Patients and methods

### Study design and randomization procedure

The present RCT was an exploratory study designed to compare the functional results and metal ion blood levels of patients who received a resurfacing total hip arthroplasty (R) against those from a conventional uncemented metal-on-metal total hip arthroplasty (C) in the short term, medium term, and long term.

**Figure 1.**
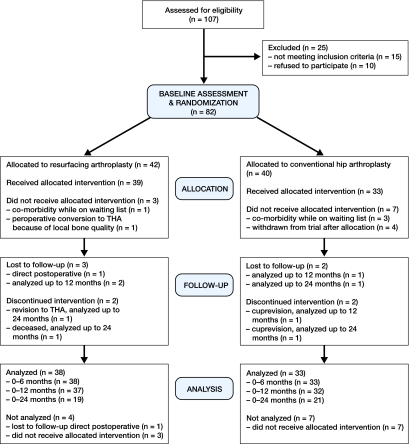
Consort statement – flow chart of participants throughout the study.

From June 2007 through January 2010, 82 patients were randomly assigned to receive one of the two hip implants (R or C). A computer-generated variable block schedule was used for randomization. The randomization list was generated by an independent statistician and the resulting treatment allocations were stored in sealed opaque envelopes. Randomization occurred at the outpatient consultation by the orthopedic surgeon at the time of planning the hip arthroplasty. The patient and surgeon could not be blinded regarding the eventual type of implant; neither of them could, however, affect the outcome of randomization. The criteria for inclusion were patients under 65 years who needed a primary hip replacement for arthritis. Patients were excluded if they had had (previous) infection of the hip or at other sites, hip fracture, avascular necrosis with collapse, osteoporotic bone mineral density, neoplasm, or renal failure. Inclusion and subsequent follow-up of patients is summarized in Figure 1. A per-protocol analysis was used in this study, because revised patients cannot be followed for metal ions. 5 patients (3 in the R group and 2 in the C group) were lost to follow-up: directly after operation (n = 1), after 12 months (n = 3), or after 24 months (n = 1). 4 patients did not participate in all follow-ups because of revision at 12 months (n = 1) or 24 months (n = 2), and 1 patient did not attend the 24-month follow-up (Figure 1). Of the randomized patients, 70 had a minimal follow-up of 12 months: 38 patients in the R group, and 32 patients in the C group.

We obtained approval from the regional ethics committee of Radboud University Nijmegen Medical Center (LTC 419-071206). All patients agreed to sign an informed consent statement. The study was performed in compliance with the Helsinki declaration and has been registered in EudraCT (2006-005610-12).

### Surgical technique

All operations were performed by 1 of 3 experienced hip surgeons using a posterolateral approach. In the R group, a resurfacing prosthesis was implanted with both components made of a cast, heat-treated solution-annealed Co-Cr alloy (Conserve plus; Wright Medical Technology, Arlington, TN). Mean resurfacing femoral head size was 48.7 mm (SD 3.5). The femoral component was cemented with low-viscosity cement after preparation of the femoral head with multiple subchondral anchor holes, and the HA coated cup was press-fitted into the acetabulum. The surgical technique has been described previously (Amstutz et al. 2006). In the C group, an uncemented tapered stem and a threaded titanium cup with a polyethylene insert with a metal liner was placed (Zweymuller Classic; Zimmer Orthopaedics, Warsaw, IN) together with a metal 28-mm head (Metasul; Zimmer Orthopaedics). Both groups received identical antibiotic prophylaxis with Cephalosporine preoperatively and 24 h postoperatively, periarticular ossification prophylaxis using Diclofenac for 3 days, and thrombosis prophylaxis with fraxiparine during hospital admission and until 6 weeks later. Patients were rehabilitated with immediate unrestricted weight bearing according to what they could tolerate (Hol et al. 2010).

### Clinical evaluation

Questionnaires that included the SF-12, Oxford hip score (OHS), and VAS implant satisfaction were filled in preoperatively and at 6, 12, and 24 months. The Harris hip score (HHS) and the University of California at Los Angeles (UCLA) activity score were assessed by an independent member of the research staff (AH).

### Blood levels of cobalt and chromium

Whole blood samples were collected preoperatively and at 3, 6, 12, and 24 months postoperatively, and assessed for cobalt and chromium concentrations. Blood was collected in metal-free Vacutainers and the first 5 mL was discarded to eliminate metal contamination from the needle. Tubes were stored at 2–8°C and sent to the toxicology laboratory of Ghent University Hospital, Belgium for analysis. The metal ion levels in serum and whole blood were determined using an inductively-coupled plasma mass spectrometer (ICP-MS). Since 9 patients (5 in the R group and 4 in the C group) with a bilateral implant (Figure 3) had double exposure to wear and thus tended to have higher blood levels of metal ions, these data are presented separately from those of unilateral implants. Extracted data from the unilateral group were considered to represent the metal ion concentration curves in the R and C groups most reliably. Following the recommendations of Daniel et al. (2007), we only report on metal ion levels in whole blood.

### Statistics

Metal ion data distributions were asymmetric and are expressed as a group median and range. Friedman's ANOVA was used for analysis. To determine the between-time differences within the groups, we used the non-parametric Wilcoxon signed-ranks test. To protect against type-1 error, a Bonferroni correction was applied. To determine differences between the two groups and between functional results, we used the Mann-Whitney U test. Symmetrical data are represented by mean and standard deviation (SD). In box plots, the outliers are represented by a dot (•) and extreme outliers (more than 3 times deviation of the interquartile range from the upper quartile) are characterized by an asterisk (*). Differences were considered statistically significant at p-values of < 0.05. Lack of information about metal ion levels and functional results because of patients not participating in all follow-ups, the small number of patients, and multiple endpoints made this an exploratory trial. The results should therefore be read as provisional. Because of the exploratory nature of the study, formal adjustment for multiplicity between endpoints was not made. All the statistical analyses were performed using SPSS software version 17.0.

## Results

The patient characteristics, without statistical significant differences between the two groups, are given in Table 1. Mean follow-up for both groups was 20 months. Of the 71 patients, we present a follow-up of 70 at 1 year and of 40 at 2 years. Mean operating time was longer for the R group, 77 min as opposed to 57 min (p < 0.001). Median blood loss was the same for the two groups.

**Table 1.** Patient characteristics

	R	C
	(n = 38)	(n = 33)
Median age in years (range)	58 (24–65)	59 (37–65)
Mean body mass index (SD)	26 (3.1)	28 (5.1)
Sex ratio (men:women)	21:17	21:12
Uni- or bilateral MoM prosthesis	33:5	29:4
Diagnosis (OA/AVN/CHD) **^a^**	35/1/2	31/0/2
Charnley category (A/B)	24/14	23/10

**^a^** OA: osteoarthritis; AVN: avascular necrosis; CHD: congenital hip dysplasia.

### Clinical evaluation

**Figure 2.**
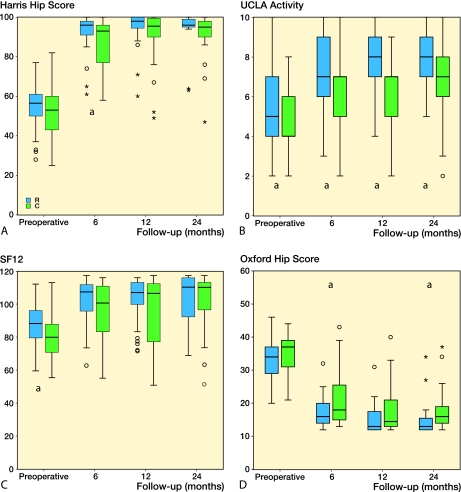
A. Boxplot of Harris hip score. B. Boxplot of UCLA activity score. C. Boxplot of SF-12. D. Boxplot of Oxford hip score. **^a ^**Significant differences between the R and C groups.

The clinical scores are summarized in Table 2 and Figure 2. In spite of the fact that we performed a randomized trial, the preoperative values of UCLA activity score and SF-12 were lower in the C group. The HHS, OHS, UCLA activity score, and SF-12 all improved after surgery in both groups (p < 0.001). This improvement in clinical scores remained stable throughout the 24-month follow-up. At 6 and 24 months, we found a better OHS in the R-group patients than in the C-group patients (p = 0.04, r = –0.33). The median UCLA activity score was better for the R-group patients at all three time points with medium effect size (6 months: p = 0.01, r = –0.30; at 12 months: p = 0.002, r =–0.38; and at 24 months: p = 0.04, r = –0.32). At 24 months, there was one negative outlier in the C group with a UCLA activity score of 2. This patient has a contralateral hip arthritis, which may explain his low activity score since his satisfaction score for the operated side was 98/100. R-group patients were more satisfied after 12 months than C-group patients (p = 0.01, r = –0.30); this difference remained but was not statistically significant at 24 months.

**Table 2.** Clinical scores and satisfaction (VAS), values are median (range)

	Preoperatively		12 months		24 months	
	R	C	R	C	R	C
	(n = 38)	(n = 33)	(n = 38)	(n = 32)	(n = 19)	(n = 21)
Harris hip score	57 (28–77)	53 (25–82)	98 (60–100)	96 (49–100)	96 (63–100)	95 (47–100)
UCLA activity	5 (2–10) **^b^**	4 (2–8) **^b^**	8 (4–10) **^b^**	7 (2–9) **^b^**	8 (5–10) **^b^**	7 (2–10) **^b^**
SF-12	88 (59–112) **^b^**	79 (55–113) **^b^**	107 (71–116)	107 (51–117)	110 (69–117)	110 (51–133)
Oxford hip score	34 (20–46)	37 (21–44)	13 (12–31)	15 (12–40)	13 (12–34) **^b^**	16 (12–37) **^b^**
VAS satisfaction	89 (49–100) **^a^**	82 (10–100) **^a^**	92 (52–100) **^b^**	85 (12–100) **^b^**	92 (37–100)	89 (15–100)

**^a^** VAS satisfaction: 6-month value.

**^b^** Significant difference between resurfacing (R) and conventional (C) hip arthroplasty (p ≤ 0.05).

Since we encountered statistically significantly better functional scores for R-group patients at some time points, including the preoperative one, we also compared the actual improvement in score between groups. In this analysis, statistically significant differences in actual improvement in the various clinical scores could no longer be detected.

### Blood levels of cobalt and chromium

**Figure 3.**
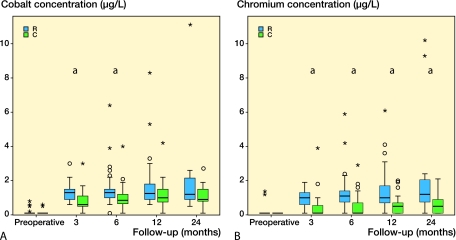
A. Boxplot cobalt concentrations of unilateral prosthesis in blood in µg/L. Two extreme outliers are not represented for clarity purposes, this concerns two R patients at 6 and 24 months with cobalt concentration of respectively 22.80 and 22.00 µg/L. B. Boxplot chromium concentrations of unilateral prosthesis in blood in µg/L. One extreme outlier was not represented for clarity purposes, this concerns a R patient at 6 months with a chromium concentration of 14.90 µg/L. **^a^** Significant differences between the R and C groups.

The concentrations of chromium and cobalt in whole blood of patients in the R and C groups for each time point are summarized in Table 3 and Figure 3. As expected, baseline preoperative chromium and cobalt concentrations were below the reference level of 1.0 µg/L for patients with a unilateral implant (both groups) (Mayo Medical Laboratories 2010). Blood cobalt and chromium levels increased (p < 0.001) in both groups after surgery until 6 months postoperatively, with stable concentrations thereafter. Cobalt concentrations were higher for R-group patients than for C-group patients only at 3 months (p < 0.001, r = –0.50) and 6 months (p = 0.006, r = –0.35). Cobalt levels stabilized after 6 months in the R group, and the initially statistically significant difference between groups could no longer be detected at 12 months (p = 0.1) and 24 months (p = 0.1). Chromium concentrations were also higher in the R group, but this time at all time points until 24 months and with a large effect size (p < 0.001, r = –0.50). We could not establish any correlation between metal ion concentration and gender, femoral component diameter, or age.

**Table 3.** Whole-blood cobalt and chromium concentrations, values (µg/L) are median (range)

	Preoperative		6 months		12 months		24 months	
	R	C	R	C	R	C	R	C
Unilateral implants								
	(n=33)	(n=29)	(n=33)	(n=29)	(n=33)	(n=28)	(n=16)	(n=17)
Co	0.1 (0.1–0.8)	0.1 (0.1–0.6)	1.3 (0.1–23) **^a^**	0.85 (0.1–4.0) **^a^**	1.25 (0.6–8.3)	1.0 (0.1–4.2)	1.2 (0.5–22)	0.9 (0.1–2.7)
Cr	0.1 (0.1–1.4)	0.1 (0.1–0.1)	1.1 (0.1–15) **^a^**	0.1 (0.1–2.9) **^a^**	1.0 (0.1–6.1) **^a^**	0.5 (0.1–2.0) **^a^**	1.2 (0.1–10) **^a^**	0.5 (0.1–2.1) **^a^**
Bilateral implants								
	(n=5)	(n=4)	(n=5)	(n=4)	(n=5)	(n=4)	(n=3)	(n=4)
Co	0.3 (0.1–1.1)	0.1 (0.1–1.8)	1.7 (1–7.9)	0.85 (0.5–2.2)	1.9 (0.9–11)	1.15 (0.8–1.3)	2.0 (0.7–6.0)	1.4 (0.7–1.8)
Cr	0.1 (0.1–0.9)	0.1 (0.1–0.8)	1.7 (0.1–3.8)	0.25 (0.1–1.5)	2.2 (0.1–4.9)	0.5 (0.1–0.8)	1.5 (0.1–2.3)	0.75 (0.6–0.8)

**^a ^**Significant difference between resurfacing (R) and conventional (C) hip arthroplasty (p ≤ 0.05).

4 patients (3 in the R group and 1 in the C group) had extremely high levels of metal ions at 12 and 24 months (Figure 3). At these time points they had good clinical scores with HHS of 98 (95–100), OHS of 13 (12–16), and a median UCLA activity score of 6.5 (6–8). These patients will be monitored closely.

The subgroup of bilateral metal-on-metal implants did not have statistically significantly higher metal ion concentrations than the unilateral group, but it should be noted that the bilateral subgroup was small (Table 3). One extreme outlier of cobalt with a concentration of 10.6 µg/L at 12 months was encountered in a male (R-group) patient with a bilateral implant. This patient had a high UCLA activity score of 9, HHS of 98, and an excellent satisfaction score of 95/100. There were no statistically significant differences between unilateral and bilateral prostheses regarding cobalt levels (p = 0.2) and chromium levels (p = 0.8) at 12 months.

### Complications

There were 3 C-group patients with recurrent dislocation, for which 2 patients had an early re-intervention with cup insert and head exchange, and there were no dislocations afterwards. In addition, 2 early deep infections were encountered in the C group without recurrence of infection after lavage. In the R group, one early revision was encountered; an early aseptic loosening from avascular necrosis of the femoral head dictated conversion to an intramedullary stem with a large femoral head. The metal acetabular cup was kept since the patient had been pain-free for 2 years and the whole-blood cobalt level was 2.3 µg/L prior to revision.

## Discussion

The most important findings of this exploratory study were that after 1 and 2 years, chromium concentrations were higher in patients with resurfacing hip arthroplasty, and cobalt concentrations were comparable with those in patients with a conventional arthroplasty. The functional results improved substantially in both groups without any major differences between groups.

The functional results—tested by validated functional scales—showed a highly significant postoperative improvement in both groups, which is in line with other studies (Pollard et al. 2006, Vail et al. 2006, Fowble et al. 2009, Mont et al. 2009, Garbuz et al. 2010, Lavigne et al. 2010, Stulberg et al. 2010, Vendittoli et al. 2010). A limitation of some of these studies was that the preoperative values of the resurfacing group were different from those in the control group (Vail et al. 2006, Fowble et al. 2009, Mont et al. 2009). In our RCT, this confounding factor was not apparent, as the preoperative HHS and OHS values were similar. In spite of the randomized nature of the present study, the preoperative UCLA activity score and SF-12 score were higher in the R-group patients. The preoperative values of the SF-12 in the C group were lower, and the mental part of the SF-12 accounted for this difference. The difference in preoperative SF-12 is difficult to explain; perhaps the C patients were less satisfied with the implant allocated to them. Obtaining the SF-12 result before informing the patient about the implant allocated to him or her could perhaps have prevented this confounding variable. This idea is also supported by our experience that inclusion for randomization proved to be extremely difficult. Generally speaking, patients tended to prefer a resurfacing arthroplasty. It was only after considerable explanation that we managed to include 82 patients. The difficulty we encountered in performing a well-designed RCT was also illustrated by the relatively high number of exclusions after randomization in the C group; 4 patients still decided to have further treatment at another hospital and withdrew from the study after having been allocated to total hip arthroplasty.

Some functional outcome scores showed a statistically significant difference in favor of the R group at certain time points. For example, satisfaction by VAS at 12 months favored the R group; however, this difference was inapparent at the 24-month follow-up. The OHS was also significantly better in the R group at 6 and 24 months, with a medium effect size, whereas there was no significant difference in the preoperative baseline levels. The UCLA activity score was significantly higher for the R group at each time point until 24 months, however, it has been noted that this difference also already applies for the preoperative scores, despite the randomization procedure. These findings correspond to those in earlier studies. From their large, retrospective comparative study Stulberg et al. (2010) reported an initial advantage in HHS from resurfacing at 6 and 12 months, but the results were comparable after 24 months. Higher UCLA activity scores after 5–7 years were also described for resurfacing in another matched cohort study with C-group patients (Pollard et al. 2006). There has only been one other RCT comparing resurfacing with conventional metal-on-metal hip arthroplasty (Vendittoli et al. 2006). The authors also reported initial UCLA activity scores in favor of the R group. However, in a recent 3–6-year follow-up report of the same study, the statistical significance of this difference was no longer apparent (Vendittoli et al. 2010). In their matched control study, Mont et al. (2009) also found a significantly better activity level after 3 years, and no significant differences in HHS and satisfaction. They claimed that the activity scores were influenced by higher baseline levels and selection bias from targeted choice of implant. Selection bias was excluded in our study by randomization; however, patient outcome can still be influenced by patients having received their “preferred” implant.

With 3 patients having a re-intervention, the percentage of early revision in this study was 4%. Of these 3 patients, 2 in the C group underwent a relatively simple insert exchange for recurrent dislocation and 1 R-group patient had a femoral component revision because of early aseptic loosening from avascular necrosis. 3 patients in the C group of 33 patients suffered a dislocation, which is an unusually high percentage. We do not have a clear explanation for this phenomenon, and do not recognize this high number from our own practice/experience. In 2 patients, a stabilizing insert exchange was performed after CT scanning had revealed proper implant positioning; no dislocations occurred after this exchange of insert.

Besides functional results as a measurement of postoperative outcome, determination of metal ion levels is becoming increasingly common after metal-on-metal arthroplasties and serves as an indicator of bearing performance and device safety (Back et al. 2005). High metal ion concentrations may lead to adverse biological reactions including local soft tissue toxicity; hypersensitivity reactions; impaired renal, endocrine, and immune function; bone loss; and risk of carcinogenesis (Shimmin et al. 2005, Keegan et al. 2007). In the present study, initially, resurfacing gave a larger increase in cobalt and chromium concentrations than a 28-mm metal bearing hip arthroplasty. After their respective run-in phases, cobalt blood levels were similar between the two groups at 12 months. Only chromium blood levels remained statistically significantly higher in the R group at all time points. Since cobalt is known to be relatively toxic compared to chromium, it is important to have established that blood cobalt levels (specifically) stabilize after a run-in phase of 6 months, and that the difference in blood levels with the C group in this study could only be established during the first 6 months of follow-up. As compared to most of the published case-controlled or retrospective reports (Back et al. 2005, Antoniou et al. 2008, Moroni et al. 2011) on the performance of several types of resurfacing implants, the metal ion levels after unilateral resurfacing in our study appeared to be rather low—with median blood levels of cobalt of 1.3 µg/L and of chromium of 1.2 µg/L. This observation may be an implant-related phenomenon.

The relationship between relatively high metal ions and the need for revision surgery after resurfacing has already been explored (De Smet et al. 2008). In spite of this recognized association between elevated cobalt and chromium levels in blood and malfunctioning implants, there is limited information about the range of acceptable metal ion concentrations and where toxicity is introduced. The best-defined reference values are the “exposure equivalent of carcinogenic substances” (EKA values) (Morgan and Schaller 1999) for industrial workers and those in the Mayo Medical Laboratories interpretive handbook (Mayo Medical Laboratories 2010). The EKA upper limits for cobalt have been defined to be 5 µg/L in whole blood and those for chromium to be 17 µg/L in erythrocytes (as no whole blood upper limits have been reported). From their own clinical series with malfunctioning resurfacing implants, De Smet et al. (2009) proposed an upper acceptable limit of 4.4 µg/L for cobalt and 5.15 µg/L for chromium in serum. The median ion levels in the present study were well below this limit, although a few outliers were encountered. Since a strong correlation between high metal ion concentrations and component wear has been established, forthcoming early revisions can still be expected in our group of patients with longer follow-up (De Smet et al. 2008).

On theoretical grounds (Smith et al. 2001), the wear of small- and large-diameter bearings—and therefore the metal ion concentrations—should be equal. In the present study, this applied to cobalt, but not to chromium. This is partially consistent with results from the literature (Antoniou et al. 2008, Moroni et al. 2008). The medium-term follow-up of Moroni et al. (2008) showed that after 5 years there were no differences in metal ion concentrations between large-diameter resurfacing hip arthroplasty and small-diameter metal-on-metal hip arthroplasty. Differences found at 3 and 6 months between R patients and C patients (28-mm) for cobalt—and at all time intervals for chromium—may be attributed to a run-in phase. Interestingly, the run-in phase of the small-diameter head in hip arthroplasty appeared to be longer, with a peak at 12 months, while the large-diameter head concentrations peaked at 6 months and stabilized thereafter.

In conclusion, we believe that the results of our study are supported by the only other published RCT comparing resurfacing with a 28-mm conventional hip arthroplasty (Vendittoli et al. 2010). The strength of our study compared to that study is that we combined clinical follow-up with prospective evaluation of metal ion levels. In addition, 2 independent RCTs with comparable outcome lead to an increase in the level of evidence of the findings. On the other hand, there are also clear limitations to our study. Inclusion of patients proved to be extremely difficult, and the number of patients available was therefore limited. Due to the limited number of patients, we can only present our data as an exploratory trial, mainly because it had insufficient power to allow us to draw firm conclusions, but also because the report deals with a short-term follow-up. Especially in the light of reports in the literature on a peak in revisions after resurfacing at 3 years of follow-up (De Smet et al. 2008), we can expect that more revisions in the R group will appear. Also, from the fact that at 24 months after surgery we encountered some clinically well functioning resurfacings with relatively high levels of metal ions, these patients may still become symptomatic in the near future and the revision rate may increase. We will continue to monitor these patients with repeated metal ion measurements and functional assessment. Longer follow-up of these two groups of patients may help us to understand the potential advantages and disadvantages of resurfacing compared to conventional arthroplasty.
